# LncRNA CASC19 Enhances the Radioresistance of Nasopharyngeal Carcinoma by Regulating the miR-340-3p/FKBP5 Axis

**DOI:** 10.3390/ijms24033047

**Published:** 2023-02-03

**Authors:** Hongxia Liu, Qianping Chen, Wang Zheng, Yuchuan Zhou, Yang Bai, Yan Pan, Jianghong Zhang, Chunlin Shao

**Affiliations:** 1 Institute of Radiation Medicine, Shanghai Medical College, Fudan University, Shanghai 200032, China; 2 School of Stomatology, Henan University, Kaifeng 475001, China

**Keywords:** nasopharyngeal carcinoma, radioresistance, lncRNA CASC19, miR-340-3p, FKBP5, autophagy

## Abstract

Radioresistance remains a serious obstacle encountered in the radiotherapy of nasopharyngeal carcinoma (NPC). Both mRNAs and non-coding RNAs (ncRNAs), including long ncRNA (lncRNA) and microRNA (miRNA), play essential roles in radiosensitivity. However, the comprehensive expression profiles and competing endogenous RNA (ceRNA) regulatory networks among lncRNAs, miRNAs, and mRNAs in NPC radioresistance are still bewildering. In this study, we performed an RNA-sequencing (RNA-seq) assay in the radioresistant NPC cells CNE2R and its parental cells CNE2 to identify the differentially expressed lncRNAs, miRNAs, and mRNAs. The ceRNA networks containing lncRNAs, miRNAs, and mRNAs were predicted on the basis of the Pearson correlation coefficients and authoritative miRanda databases. In accordance with bioinformatic analysis of the data of the tandem mass tag (TMT) assay of CNE2R and CNE2 cells and the gene chip assay of radioresistant NPC samples in pre- and post-radiotherapy, the radioresistance-related signaling network of lncRNA CASC19, miR-340-3p, and FKBP5 was screened and further verified using an RT-qPCR assay. CASC19 was positively associated with FKBP5 expression while negatively correlated with miR-340-3p, and the target binding sites of CASC19/miR-340-3p and miR-340-3p/FKBP5 were confirmed using a dual-luciferase reporter assay. Moreover, using an mRFP–GFP–LC3 maker, it was found that autophagy contributed to the radioresistance of NPC. MiR-340-3p inhibition or FKBP5 overexpression could rescue the suppression of autophagy and radioresistance induced by CASC19 knockdown in CNE2R cells. In conclusion, the CASC19/miR-340-3p/FKBP5 network may be instrumental in regulating NPC radioresistance by enhancing autophagy, which provides potential new therapeutic targets for NPC.

## 1. Introduction

Nasopharyngeal carcinoma (NPC), a unique type of head and neck cancer arising from the nasopharynx, has remarkable geographic and racial distribution worldwide. NPC has a high prevalence in Southeast Asia and southern China [1,2]. Due to its unique anatomical location and relatively high radiosensitivity, radiotherapy has become the first choice for the treatment of NPC [3]. However, approximately 30% of NPC patients eventually develop recurrence or/and distant metastasis due to radioresistance [4]. Therefore, it is crucial to further investigate the molecular mechanism underlying the residual NPC cells after irradiation to avoid radiotherapy failure.

At present, many non-coding RNAs (ncRNAs) have been identified and demonstrated to play vital roles in multiple physiological and pathological processes. Based on the size, these ncRNAs are subdivided into small ncRNAs (<200 nt, such as microRNAs) and long ncRNAs (lncRNAs) (>200 nt) [5]. Dysregulated lncRNAs are involved in tumorigenesis and even the radioresistance of NPC. Therefore, some lncRNAs, including PVT1 [6], DANCR [7], MALAT1 [8], and FAM225A [9], have been confirmed as oncogenes in NPC. It was reported that targeting PVT1 might be a potential strategy for improving the curative effect of NPC after radiotherapy [10]. A recent study revealed that lncRNA linc00312 suppressed radioresistance by targeting DNA-PKcs and impairing DNA damage repair in NPC [11]. MiRNAs, composed of 19–25 nucleotides, are a kind of small endogenous non-coding RNAs. With respect to NPC, several miRNAs such as miR-20a-5p [12], miR-BART4 [13], and miR-483−5p [14] conferred radioresistance, while other miRNAs, including miR-450b-5p [15], miR-34c [16], and miR-24-3p [17], contributed to radiosensitivity.

Recently, the network of competing endogenous RNA (ceRNA) has been regarded as an important mechanism involved in post-transcriptional gene translation regulation [18], and the activity of ceRNAs could alter the function of the target miRNA [19]. Emerging evidence has uncovered that the ceRNAs play important roles in the development of many kinds of tumors, including NPC. For instance, as a ceRNA, lncRNA MEG3 upregulated the AKT2 expression by sponging miR-200b-3p in the osteosarcoma [20]. LncRNA PVT1 promoted the gemcitabine resistance of pancreatic cancer by activating the Wnt/β-catenin and autophagy pathways through modulating miR-619-5p/Pygo2 and miR-619-5p/ATG14 axes [21]. LINC00518 regulated glycolysis through miR-33a-3p /HIF-1α negative feedback loop and induced radioresistance in melanoma [22]. For NPC, the lncRNA PTPRG-AS1, as a molecular sponge of microRNA-194-3p, indirectly regulated radiosensitivity [23], and the lncRNA FAM225A promoted tumorigenesis and metastasis by sponging miR-590-3p/miR-1275 and upregulating ITGB3 [9]. However, so far, the comprehensive expression profiles of lncRNA, miRNA, and mRNA, as well as the underlying ceRNA regulatory networks in the radioresistance of NPC, have not been disclosed yet.

In this study, we identified the differentially expressed lncRNAs, miRNAs, and mRNAs (DElncRNAs, DEmiRNAs, and DEmRNAs) between radiosensitive NPC cells CNE2 and its radioresistant cells CNE2R through RNA sequencing, and the ceRNA networks of screened lncRNA, miRNA, and mRNA were predicted with authoritative miRanda database and further confirmed using RT-PCR. On the other hand, autophagy, as a conservative lysosome-mediated intracellular degradation system, is critical for maintaining cellular homeostasis. It was reported that autophagy had a positive correlation with radioresistance in NPC [24]. Our previous study has found that the high expression of lncRNA CASC19 (abbreviated as CASC19, hereafter) enhanced the radioresistance of NPC by promoting autophagy [25]. This mechanism study revealed for the first time that CASC19 could function as a ceRNA to promote autophagy and hence increase the radioresistance of NPC by suppressing miR-340-3p and increasing the expression of mRNA FKBP5, which might provide a new regulatory target of improving the efficacy of NPC radiotherapy.

## 2. Results

### 2.1. Expression Profiles of lncRNAs, miRNAs, and mRNAs in NPC Cells

**Figure 1:**
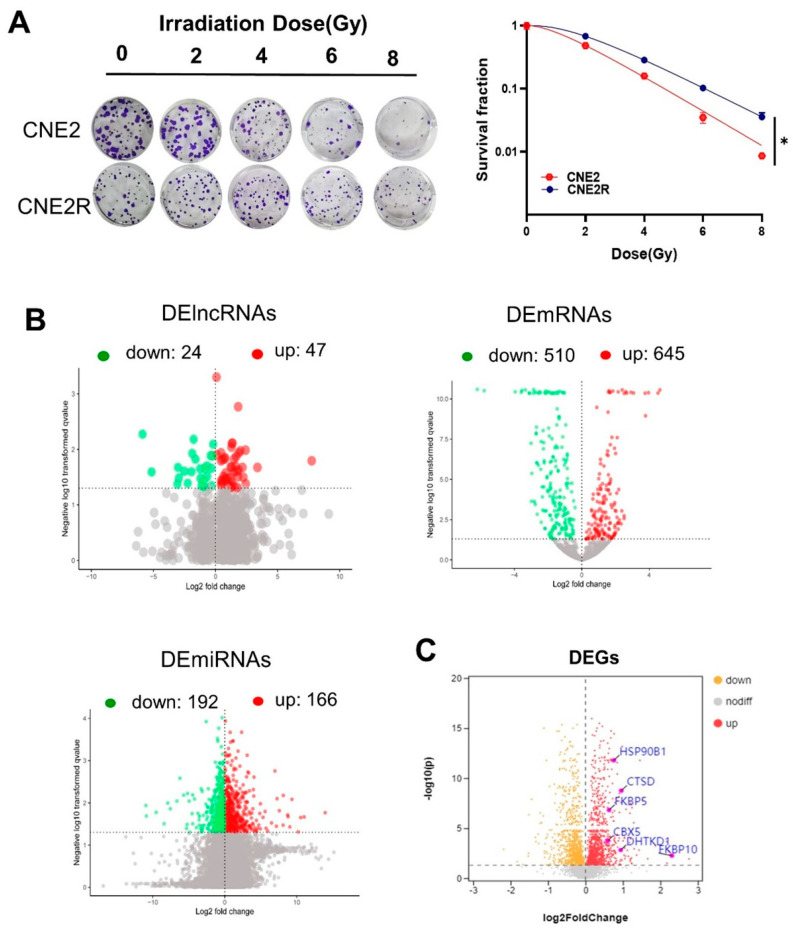
RNA profile analyses of CNE2 and its paired radioresistance CNE2R cells: (**A**) colony formation assay was performed to evaluate the radiosensitivity of CNE2 and CNE2R cells. * *p* < 0.05 between indicated groups; (**B**) volcano plots of the DElncRNAs, DEmRNAs, and DEmiRNAs between CNE2 and CNE2R cells based on RNA−seq assay; (**C**) volcano plot of differentially expressed genes (DEGs) between CNE2 and CNE2R cells based on TMT assay.

We previously successfully established a radioresistant cell line (named CNE2R) from human NPC cells CNE2 [25]. In comparison with CNE2 cells, CNE2R cells had acquired radioresistance (Figure 1A). Then, the RNA sequences of these two cell lines, including lncRNAs, miRNAs, and mRNAs, were acquired using the Illumina Hiseq2000 platform. It was found that there were 71 DElncRNAs (47 upregulation and 24 downregulation), 358 DEmiRNAs (166 upregulation and 192 downregulation), and 1155 DEmRNAs (645 upregulation and 510 downregulation) between CNE2R and CNE2 cells (Figure 1B). Moreover, the differential expressed genes (DEGs) between CNE2 and CNE2R cells were analyzed using a TMT quantitative proteomic assay, and the volcano plot of these DEGs is shown in Figure 1C.

### 2.2. CeRNA Networks were Constructed on the Basis of Screened mRNAs and CASC19

**Figure 2:**
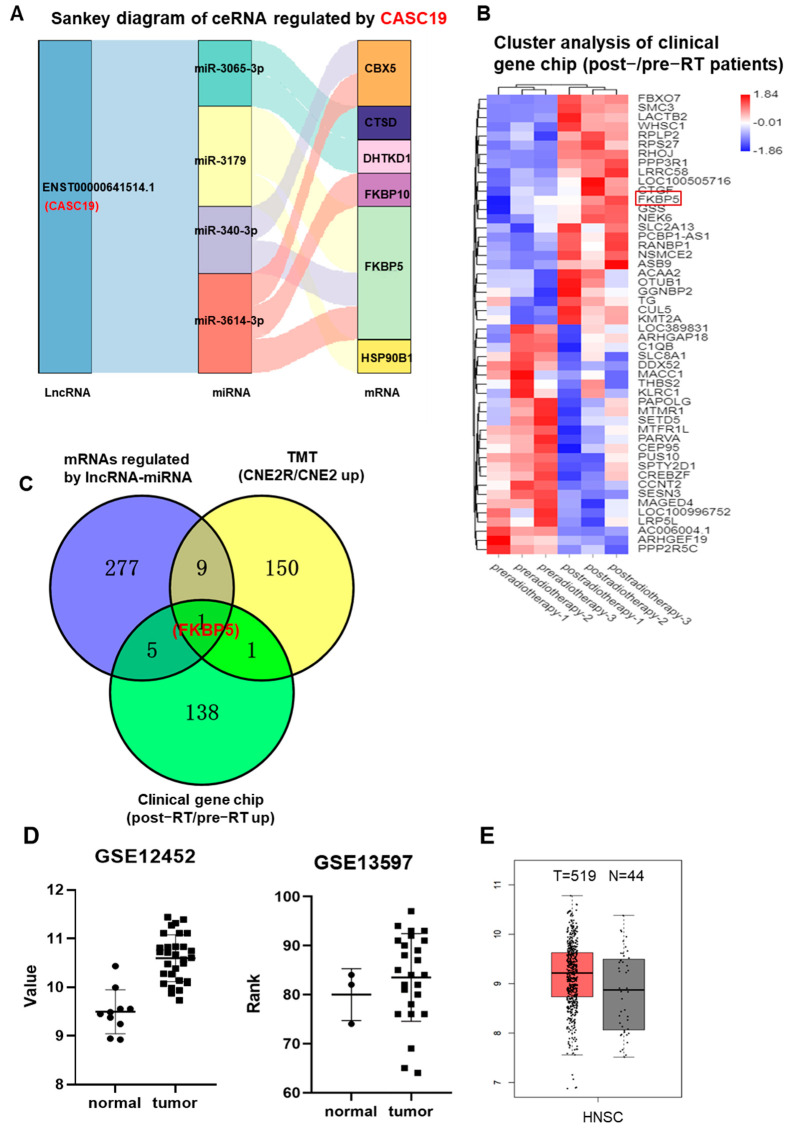
CASC19−related ceRNA networks were constructed on the basis of mRNAs screen: (**A**) CASC19−related ceRNA networks of 6 common upregulated genes (CBX5, CTSD, FKBP5, FKBP10, DHTKD1, and HSP90B1) based on the intersection of RNA−seq and TMT assays; (**B**) the heatmap of DEGs between tumor samples of NPC radioresistant patients before and after radiotherapy (n = 3); (**C**) Venny chart of the intersection of the common genes among the upregulated mRNAs regulated by lncRNAs-miRNAs in CNE2R cells (RNA−seq assay), the upregulated genes in CNE2R cells compared with CNE2 cells (TMT assay), and the upregulated genes in the patients post-radiotherapy compared with pre-radiotherapy (clinic gene chip assay). The intersection only contains one gene FKBP5; (**D**) the expressions of FKBP5 in the tumor tissues of NPC and its adjacent normal tissues (data from GEO databases of GSE12452 and GSE13597); (**E**) the expressions of FKBP5 in the head and neck squamous cell carcinoma (HNSC) and its adjacent normal tissues (data from GEPIA).

Our previous study demonstrated that CASC19 contributed to the poor prognosis and radioresistance of NPC [25]. To know the potential targets of CASC19, we established CASC19-related ceRNA networks based on the above DElncRNAs, DEmiRNAs, DEmRNAs, and DEGs. As a result, six mRNAs (FKBP5, CBX5, CTSD, DHTKD1, FKBP10, and HSP90B1) and four miRNAs (miR-340-3p, miR-3065-3p, miR-3179, and miR-3614-3p) regulated by CACS19 were included in the ceRNA networks, shown in a Sankey diagram (Figure 2A). Meanwhile, gene chip analysis gave the heat map of the distribution of DEGs between the tumor samples of three NPC radioresistant patients before and after radiotherapy (Figure 2B), and a total of 292 aberrantly expressed genes were obtained. Only one commonly high-expressed gene, FKBP5, was identified in the cross-section among the multi-omic analyses of the above RNA-seq, TMT, and gene chip (Figure 2C). Further analyses using GSE12452 and GSE13597 datasets in the GEO database demonstrated that the expression of FKBP5 was upregulated in NPC tissue in comparison with normal tissue (Figure 2D). Analysis of the GEPIA database also demonstrated that the expression of the FKBP5 gene was obviously upregulated in 519 HNSC (head and neck squamous cell carcinoma) tissues compared with that in 44 normal tissues (Figure 2E). Therefore, the high expression of FKBP5 was probably an important prognostic marker of NPC radioresistance. Moreover, it was reported that miR-340 could be decreased by ionizing radiation (IR) [26]. Hence, to demonstrate the feasibility and value of the constructed ceRNA networks, we chose the CASC19/miR-340-3p/FKBP5 axis for further validation.

### 2.3. Expressions of CASC19, FKBP5 mRNA and miR-340-3p in NPC Cells

**Figure 3:**
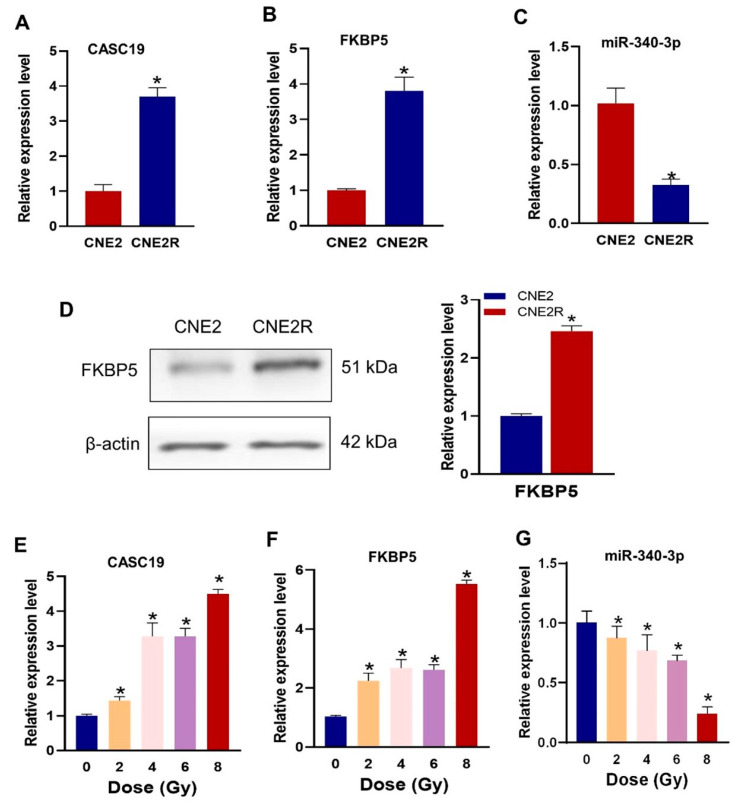
The expressions of CASC19, miR-340-3p, and FKBP5 in CNE2 and CNE2R cells: (**A**–**C**) the expressions of CACS19, FKBP5, and miR-340-3p detected using RT-PCR. * *p* < 0.05 compared with CNE2 cells; (**D**) the expression of FKBP5 protein detected with Western blot assay. * *p* < 0.05 compared with CNE2 cells; (**E**–**G**) the expressions of CASC19, FKBP5, and miR-340-3p in CNE2 cells irradiated with different doses. * *p* < 0.05 compared with nonirradiated control.

We then examined the difference in the expression levels of CASC19, FKBP5 mRNA, and miR-340-3p in radiosensitive and radioresistant NPC cell lines. It was confirmed that CASC19 and FKBP5 were significantly highly expressed (Figure 3A,B), and miR-340-3p was lowly expressed in CNE2R cells, compared with CNE2 cells (Figure 3C), indicating that CASC19 and FKBP5 had a negative correlation with miR-340-3p. Western blot assay also demonstrated that the FKBP5 protein level in CNE2R cells was higher than that in CNE2 cells (Figure 3D). Moreover, the expressions of these ceRNAs could be altered via irradiation in a dose-dependent manner. The RT-PCR assay showed that the expressions of CASC19 and FKBP5 generally increased with irradiation dose (Figure 3E,F), but the expression of miR-340-3p decreased with irradiation dose (Figure 3G).

### 2.4. CASC19 Negatively Regulated miR-340-3p to Promote Autophagy and Enhance Radioresistance

**Figure 4:**
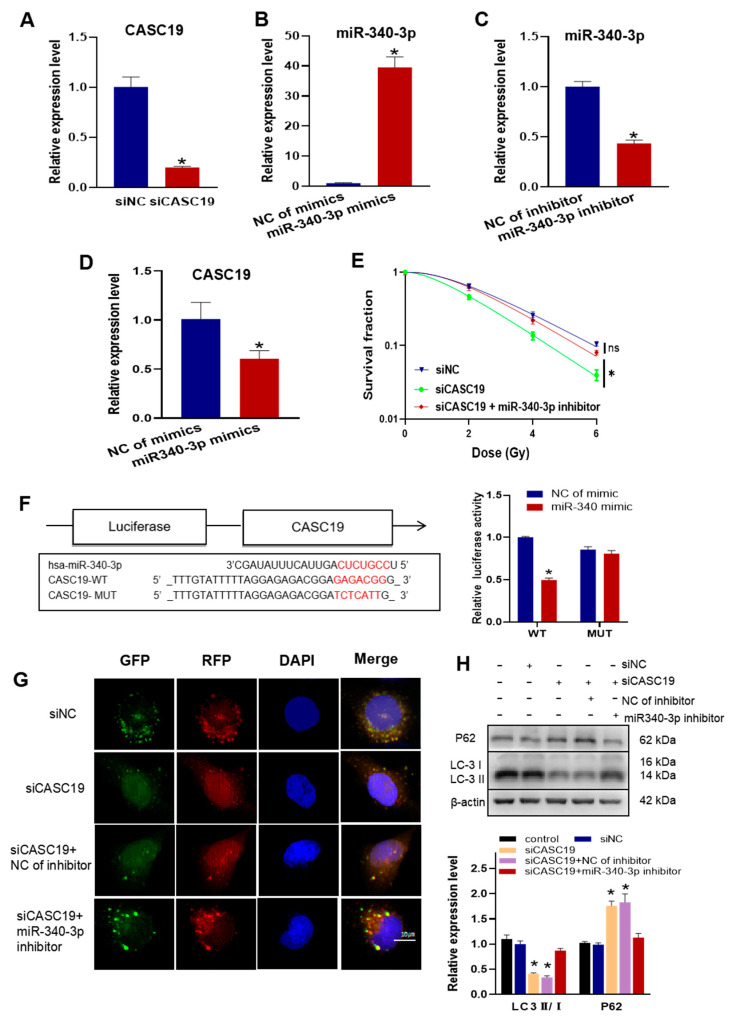
CASC19 enhanced autophagy and promoted the radioresistance of NPC by negatively regulating miR-340-3p: (**A**) verification of the interference efficiency of CASC19 smart silencer (siCASC19) in CNE2R cells; (**B**,**C**) the expression of miR-340-3p in CNE2R cells after transfection of miR-340-3p mimics (**B**) or miR-340-3p inhibitor (**C**); (**D**) the expression of CASC19 in CNE2R cells after transfection of miR-340 mimics; (**E**) survival curves of irradiated CNE2R cells transfected with siCASC19, miR-340 inhibitor, or its control; (**F**) prediction of the target binding sites of CASC19 and miR-340-3p was verified using the dual-luciferase reporter assay; (**G**) autophagy assay using mRFP-GFP-LC3 in CNE2R cells transfected with siCASC19, miR-340 inhibitor, or their controls; (**H**) Western blot assay of P62 and LC3 proteins in CNE2R cells transfected with siCASC19, miR-340 inhibitor, or their controls. * *p* < 0.05 compared with corresponding control or between indicated groups.

To know the relationship between the functions of CASC19 and miR-340-3p in radioresistance, we interfered in CNE2R cells with the CASC19 smart silencer (siCASC19), miR-340-3p mimics, miR-340-3p inhibitor, and their negative controls with high efficiencies (Figure 4A–C). It was found that the expression of CASC19 was significantly downregulated by miR-340-3p mimics (Figure 4D), and the transfection of CNE2R cells with siCASC19 decreased cell survival after irradiation, which was rescued by the miR-340-3p inhibitor (Figure 4E). Therefore, CASC19 might promote the radioresistance of NPC cells by regulating miR-340-3p. The bioinformatic analysis predicted that miR-340-3p targeted the 3′ UTRs of CASC19 with complementary binding sites (Figure 4F). Then, we constructed luciferase reporter plasmids containing wild-type (WT) CASC19 or mutant-type (MUT) CASC19 and co-transfected them with miR-340-3p mimics into CNE2R cells. It was found that the luciferase activity of the reporter was reduced under the situation of MUT-CASC19 but had no alteration under WT-CASC19 (Figure 4F), demonstrating that CASC19 serves as a sponge for miR-340-3p.

Furthermore, to determine whether CASC19 contributes to the autophagy-regulated radioresistance of NPC cells by negatively regulating miR-340-3p, we transfected siCASC19 and/or miR-340-3p inhibitor together with an autophagy indicator Ad-mRFP-GFP-LC3 into radioresistant CNE2R cells. As a result, siCASC19 reduced the number of autophagic LC3 spots in CNE2R cells, which was also rescued by the miR-340-3p inhibitor (Figure 4G). In addition, the ratio of LC3II/I was significantly reduced, and the expression of P62 was increased in CNE2R cells by siCASC19 transfection (Figure 4H), but these alterations did not occur when the cells were transfected with siCASC19 together with the miR-340-3p inhibitor. Accordingly, the functions of CASC19 in radioresistance and autophagy relied on its role of acting as the sponge of miR-340-3p.

### 2.5. CASC19 Positively Regulated the Expression of FKBP5 through miR-340-3p

**Figure 5:**
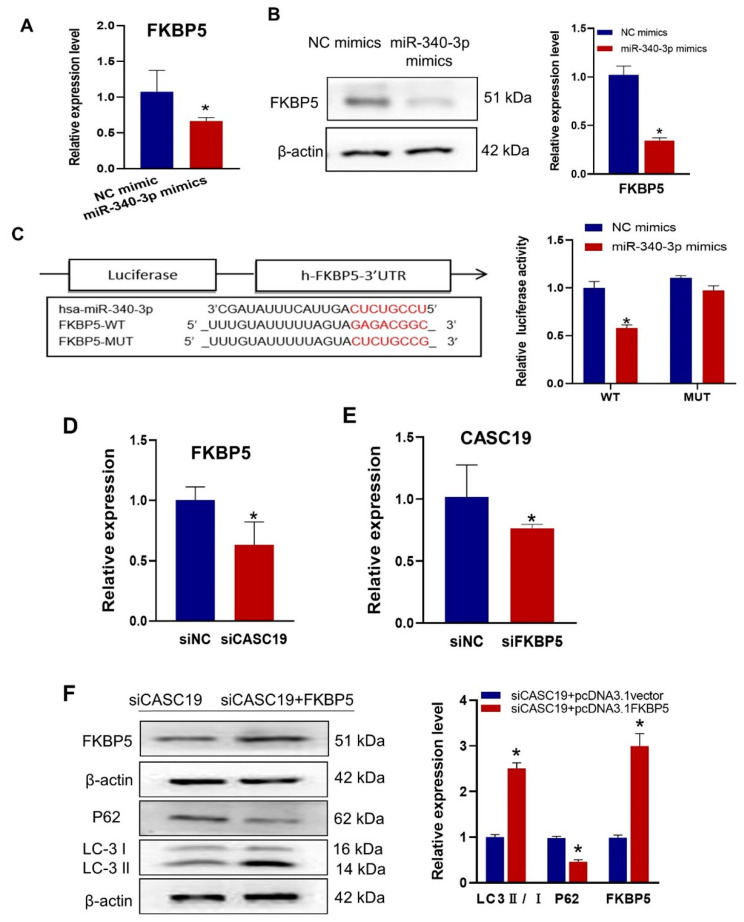
CASC19 positively regulated the expression of FKBP5 through miR-340-3p: (**A**,**B**) RT-qPCR (**A**) and Western blot (**B**) assay of the expression of FKBP5 in CNE2R cells transfected with miR-340-3p mimics or its control; (**C**) prediction of the target binding sites of FKBP5 and miR-340-3p was verified with the dual-luciferase reporter assay; (**D**) the expression of FKBP5 in CNE2R cells with or without transfection of siCASC19; (**E**) the expression of CACS19 in CNE2R cells with or without transfection of siFKBP5; (**F**) Western blot assay of FKBP5, P62, and LC3 protein in CNE2R cells co-transfected with siCASC19 and FKBP5 overexpression plasmid. * *p* < 0.05 compared with the corresponding control.

It was found that the mRNA and protein expression levels of FKBP5 were significantly decreased in CNE2R cells after transfection with miR-340-3p mimics (Figure 5A,B). Using the bioinformatic prediction tools of miRanda, RNAhybrid, and TargetScan databases, it was demonstrated that miR-340-3p could target the 3′ UTR of FKBP5 mRNA with complementary binding sites, which was validated with the luciferase reporter gene assay (Figure 5C). Further investigation showed that the downregulation of CASC19 positively affected FKBP5 expression in CNE2R cells (Figure 5D), and the expression of CASC19 was decreased after knocking down FKBP5 (Figure 5E). To verify the FKBP5-dependent radioresistance function of CASC19, we transfected CNE2R cells with siCASC19 or its scrambled vector together with the FKBP5-overexpression vector or its corresponding empty vector. Our results showed that the overexpression of FKBP5 increased the ratio of LC3 II/I but decreased the expression of P62 (Figure 5F). Taken together, these findings demonstrated that CASC19 contributed to the radioresistance of NPC cells via the CASC19/miR-340-3p/FKBP5 regulatory axis.

### 2.6. Inhibition of FKBP5 Increased Radiosensitivity by Inhibiting Autophagy

**Figure 6:**
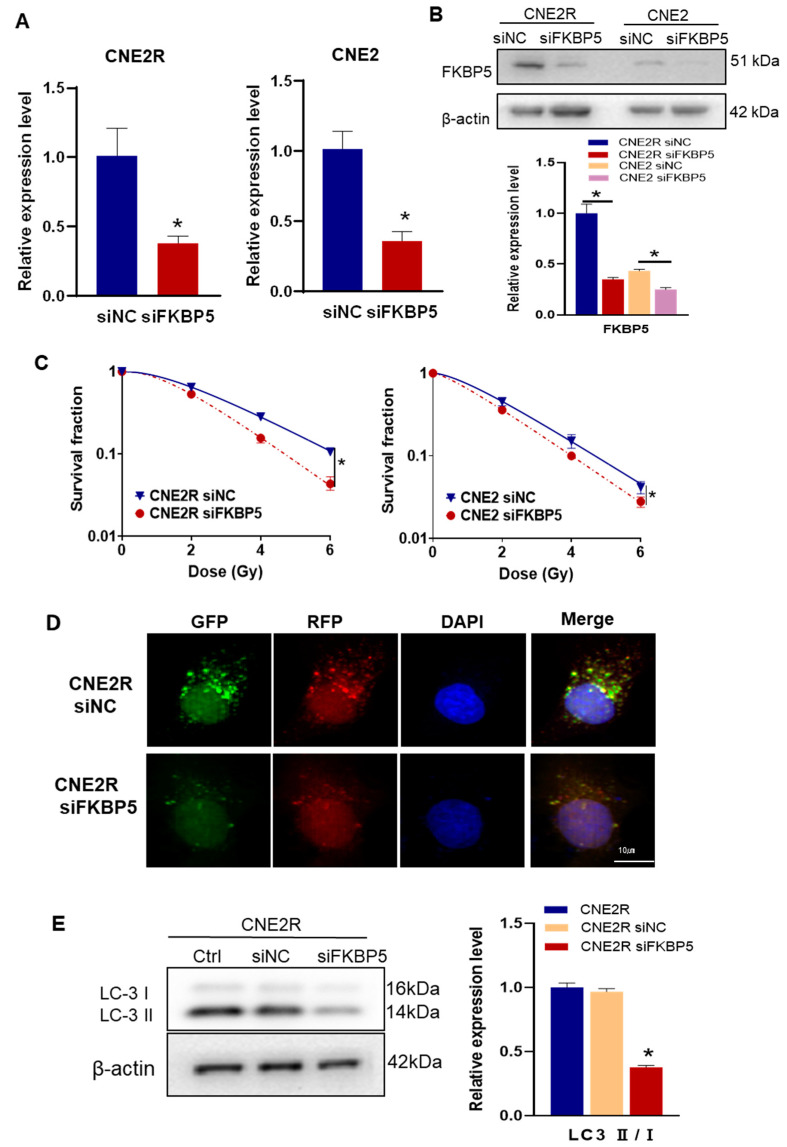
Knockdown FKBP5 enhanced the radiosensitivity of NPC cells by inhibiting autophagy: (**A**,**B**) RT-qPCR (**A**) and Western blot (**B**) assay of the expression of FKBP5 in CNE2R and CNE2 cells transfected with siFKBP5; (**C**) colony formation assay was performed to evaluate the influence of siFKBP5 on the survival fractions of CNE2R and CNE2 cells after irradiation; (**D**) fluorescence images of CNE2R cells transfected with siFKBP5 and mRFP-GFP-LC3-tagged adenovirus (×40); (**E**) Western blot assay of LC3 proteins in CNE2R cells after transfection with siFKBP5. * *p* < 0.05 compared with corresponding siNC or between indicated groups.

It has been reported that FKBP5 can promote autophagy and increase the radioresistance of melanoma [27]. To know the regulation function of FKBP5 in the radiosensitivity of NPC, we transfected CNE2R and CNE2 cells with siFKBP5 and its negative control with a high transfection efficiency (Figure 6A,B). After siFKBP5 interference, the survival fractions of irradiated NPC cells were significantly decreased, which was considerably profound in CNE2R cells (Figure 6C). To determine whether FKBP5 contributes to the autophagy-regulated radioresistance of NPC cells, we transfected siFKBP5 and Ad-mRFP-GFP-LC3 into radioresistant CNE2R cells and found that the number of autophagic LC3 spots in the siFKBP5 group sharply decreased in comparison with the negative control of siRNA (Figure 6D). In line with this finding, the suppression of FKBP5 also decreased the ratio of LC3II/I in CNE2R cells (Figure 6E). These results demonstrated that siFKBP5 enhanced the radiosensitivity of NPC cells by inhibiting autophagy.

## 3. Discussion

Radioresistance is a serious obstacle encountered in NPC treatment. During the past few decades, besides mRNAs, an increasing number of ncRNAs, including miRNA and lncRNA, have been demonstrated to play an essential role in the radioresistance of tumors. Actually, some pivotal mRNAs and ncRNAs have been reported to be involved in NPC radioresistance [28,29]. Moreover, several lines of evidence have shown that some ncRNAs can form ceRNA networks to competitively regulate the expression of critical coding genes [18]. Thus far, there have been many reports on the whole transcriptome expression profiles and related ceRNA regulatory networks in carcinogenesis and tumor chemoresistance [30,31]. However, there are few studies in the literature about the expression profiles of ncRNAs and related-ceRNA regulatory networks for the radioresistance of NPC. Therefore, clarifying the ceRNA regulatory network can improve the understanding of the mechanism of NPC radioresistance.

CASC19 is located on the 8q24 region of the chromosome and is referred to as an “oasis” of non-coding RNA [32]. Previous studies demonstrated that CASC19 was involved in clear cell renal cell carcinoma (ccRCC) [33], non-small cell lung cancer (NSCLC) [34], glioma [35], and pancreatic cancer [36]. Moreover, CASC19 could accelerate chondrocyte apoptosis and proinflammatory cytokine production and exacerbate osteoarthritis development by regulating the miR-152-3p/DDX6 axis [37]. Evidence suggested that the aberrantly expressed CASC19 was associated with chemotherapeutic drugs [38]. Notably, our previous study revealed that CASC19 contributed to NPC radioresistance [25]. As the prior function of CASC19 was mainly through the indirect adsorption of miRNAs, which affected its downstream target genes [33,34,35,36,37], we analyzed the RNA-seq of CNE2R and its parental CNE2 cells. Based on the analyses of the expression profiles of lncRNA, miRNA, and mRNA (Supplementary Tables S1–S3), as well as the TMT data of NPC cells (CNE2R vs. CNE2) (Supplementary Table S4) and the gene chip data of clinical NPC samples (Supplementary Table S5), we proposed that a novel ceRNA network of CASC19/miR-340-3p/FKBP5 contributed to the radioresistance of NPC. Currently, miR-340 is widely regarded as a tumor suppressor in a variety of cancers. For example, miR-340 suppressed cell proliferation, promoted cell apoptosis, and inhibited cell migration and invasion in gastric cancer [39,40]. In ovarian cancer, miR-340 suppressed cell metastasis by targeting FHL2 [41]. Moreover, miR-340 could target BMI1, and the ectopic upregulation of BMI1 partially reversed the effect of miR-340 on the growth and migration of colorectal cancer [42]. In addition, several other studies found that miR-340 also acted as a tumor suppressor in glioblastoma [43,44], breast cancer [45], and non-small cell lung cancer [46]. In particular, the radiation-regulated miR-340/429/IL-4/β-catenin/Stat6 signaling axis effectively enhanced tumor progression and metastasis of human carcinomas [26]. Our study demonstrated that CASC19 could act as the sponge of miRNAs and it downregulated the intracellular level of miR-340-3p and thus influenced cell autophagy and radioresistance.

As a downstream target gene of miR-340-3p, FKBP5 was identified using the bioinformatic analyses of RNA-seq, TMT, and gene chip assays of cells and clinic samples. It was found that FKBP5 could mediate the effect of the CASC19/miR-340-3p axis on autophagy and the radioresistance of NPC cells. Previous studies have shown that FKBP5, also known as FK506 binding protein 5, is a member of the immunoaffinity protein family and a modulator of the Hsp90 chaperone protein and glucocorticoid receptor [47]. Several lines of evidence have revealed its functions in cancer biology [48]. Particularly, FKBP5 played an essential role in the resistance of melanoma to radiotherapy [27]. On the other hand, FKBP5 has also been proven to be a potent inducer of autophagy [49,50,51]. For example, FKBP5 could bind to BECN1, a key regulator of autophagy, and change its phosphorylation and protein expression levels, thereby promoting autophagy. In agreement with these findings, our data also disclosed that FKBP5 contributed to radioresistance by promoting autophagy in NPC cells. FKBP5 was also confirmed to be the target gene of miR-340-3p. Furthermore, we identified that CASC19 and FKBP5 presented a positive correlation, indicating that CASC19 increased radioresistance by enhancing autophagy through the miR-340-3p/FKBP5 axis.

**Figure 7:**
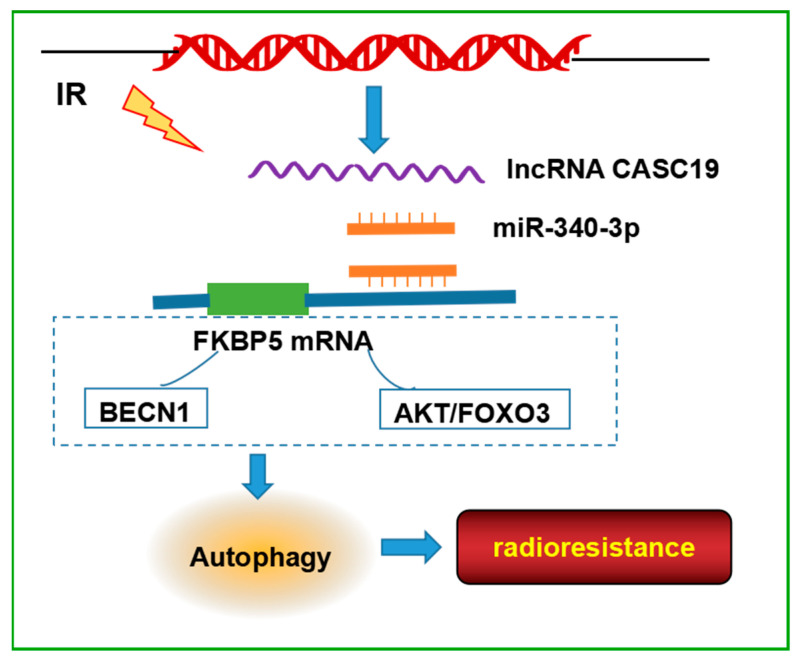
Schematic model of the pathways regulated the radioresistance of NPC cells. CASC19, as a competing endogenous RNA, bound to miR-340-3p via the lncRNA sponging mechanism and abolished the suppression of FKBP5 expression caused by miR-340-3p. FKBP5 may bind with BECN1 or regulate AKT/FOXO3 pathway, resulting in an increase in autophagy, thereby enhancing the radioresistance of NPC. The signaling pathways in the dashed box are derived from studies in the literature.

Taken together, this study presented the comprehensive analysis of the lncRNA, miRNA, and mRNA expression profiles between radioresistance and radiosensitive NPC cells and built a novel ceRNA regulatory network, CASC19/miR-340-3p/FKBP5, as a key player in modulating the radiosensitivity of NPC cells by promoting autophagy (Figure 7). These results improved the knowledge of the mechanism of NPC radioresistance and might provide potential molecular targets for increasing the efficacy of NPC radiotherapy.

## 4. Materials and Methods

### 4.1. Cell Culture and Irradiation

Human NPC cell line CNE2 was purchased from Shanghai Cell Bank (Shanghai, China). The radioresistant CNE2R cell line was previously established from its parental CNE2 cells by fractionated irradiation with a total dose of 60 Gy [25]. These cells were cultured with RPMI-1640 medium (Gibco, Hangzhou, China) supplemented with 10% fetal bovine serum (Gibco Invitrogen, Grand Island, NY, USA), 1% penicillin–streptomycin (Gibco) and maintained in a humidified atmosphere of 5% CO_2_ at 37 °C. NPC cells were irradiated with different doses using an X-ray irradiator (12 mA, 2 mm aluminum filtration; X-RAD 320, Precision X-Ray Inc., North Branford, CT, USA) at a dose rate of 0.883 Gy/min.

### 4.2. Colony Formation Assay

The radiosensitivities of NPC cell lines were assessed using a colony formation assay. Logarithmic growing cells were irradiated with different doses (2, 4, 6, and 8 Gy) of γ-rays (137-Cs, Gammacell-40, MDS Nordion, Ottawa, ON, Canada) at room temperature, cultured for about 10 days to form colonies, and then fixed and stained with crystal violet. Colonies consisting of more than 50 cells were counted for survival fraction. The cell survival curve (SF) was fitted with the single-hit multitarget model as SF = 1 − (1 − exp (−k × D))^N^.

### 4.3. RNA Isolation for Sequencing Analysis

The total cellular RNA was extracted using TRIzol reagent (Invitrogen, San Diego, CA, USA) according to the manufacturer’s instructions. DElncRNAs, DEmRNAs, and DEmiRNAs between the indicated cell lines were assessed using the DEGseq R package as previously described [29]. The transcript with Pval < 0.01 was set as the threshold for significant differences. Sequencing libraries for miRNAs were generated using NEBNext^®^ Multiplex Small RNA Library Prep Set for Illumina^®^ (New England Biolabs, Ipswich, MA, USA) following the manufacturer’s recommendation. All sequencing programs and analyses were performed by Novogene Company (Beijing, China).

### 4.4. TMT Quantitative Proteomic Analysis

Total proteins were extracted from CNE2 and CNE2R cells, and their concentrations were detected with a BCA kit according to the manufacturer’s instructions. A quantity of 0.2 mg of protein from each sample was used for TMT analysis. A high-resolution mass spectrometer Q Exactive plus (Thermo Fisher Scientific) was used to perform the quantitative proteomics analysis. The MS/MS data were performed with the Maxquant search engine (v.1.5.2.8).

### 4.5. Gene Chip Detection of Tumor Tissues of NPC Patients

Formalin-fixed paraffin-embedded (FFPE) NPC samples of three radioresistant patients pre- and post-radiotherapy were applied for gene chip detection (Bohao Biotechnology Co., Shanghai, China). According to the Declaration of Helsinki, all samples were obtained after ethical approval with the informed consent of patients receiving treatment at the Nanfang Hospital of Southern Medical University (Guangzhou, China).

### 4.6. Construction of CASC19-Associated ceRNA Network

To construct a radioresistance-related lncRNA-associated ceRNA network, we first performed RNA-seq analysis to screen the DEmiRNAs that may be involved in radioresistance [30]. Secondly, we constructed the lncRNA–miRNA–mRNA interaction network to predict the ‘sponge’ function of lncRNA. According to the expression levels of lncRNAs, miRNAs, and mRNAs, the Pearson correlation coefficients between miRNA and mRNA as well as miRNA and lncRNA were calculated, where the negative correlation coefficient > 0.85 was selected for further analyses using the miRanda database to obtain the ceRNA network. Moreover, applying the above TMT data and the gene chip data, we utilized the paired dysregulated lncRNA, miRNA, and mRNA expression profiles to construct the CASC19-associated ceRNA network involved in radioresistance using the R and Cytoscape software.

### 4.7. Real-Time PCR Assay of RNA

After the extraction of total RNA from NPC cells using a TRIzol reagent, the cDNA was synthesized from 1 μg of the total RNA using a PrimeScript RT Reagent Kit with gDNA Eraser (Takara Biotechnology, Co., Ltd., Dalian, China), and then it was amplified using real-time qPCR with Ultra SYBR Mixture (Low ROX) (CoWin Biosciences, Beijing, China) on an MX3000P platform with the internal control of housekeeping gene β-actin. MiR-340-3p was detected using a Mir-X miRNA qRT-PCR TB Green Kit (Takara Biotechnology, Co., Ltd., Dalian, China) with U6 RNA as an internal control. All primers were synthesized by Sangon (Shanghai, China) with the below sequences. For the CASC19 gene, the forward primer was 5′-TTT AGC CTG CAT AGG ACC CTC-3′, and the reverse primer was 5′-GTC TGG TCA AAT TAC AAT CAG TTG G-3′. For the β-actin gene, the forward primer was 5′-CAT GTA CGT TGC TAT CCA GGC-3′, and the reverse primer was 5′-CTC CTT AAT GTC ACG CAC GAT-3′. For miR-340-3p, the forward primer was 5′-CGC GTC CGT CTC AGT TAC TTT ATA GC-3′. For U6, the forward primer was 5′-TGG AAC GCT TCA CGA ATT TGC G-3′, and the reverse primer was 5′-GGA ACG ATA CAG AGA AGA TTA G-3′. For the FKBP5 gene, the forward primer was 5′-TAT GGC TCG GCT GGC AGT CTC-3′, and the reverse primer was 5′-CCC TCT CCT TTC CGT TTG GTT CTC-3′.

### 4.8. Western Blotting Assay

Total proteins were extracted from NPC cells with an ice-cold RIPA lysis buffer (Beyotime Biotechnology, Shanghai, China), isolated by SDS–PAGE, and transferred onto 0.22 μm PVDF membrane (Immobilon-P, Millipore Corporation). The membrane was blocked for 2 h with 5% nonfat milk and then incubated with primary antibodies overnight at 4 °C. After washing with 0.05% Tris-buffered saline/Tween (TBST) well, the membrane was incubated with HRP-conjugated secondary antibodies for 2 h. Then, the proteins were visualized with an ECL Chemiluminescence Kit (Millipore, St. Louis, MO, USA) according to the manufacturer’s instructions, and the protein band images were analyzed with the Bio-Rad ChemiDoc XRS system.

### 4.9. siRNA Transfection

NPC cells were transferred with siRNA FKBP5 (siFKBP5, target sequence: 5′-CCC UCG AAU GCA ACU CUC UTT-3′), miR-340 inhibitor (5′-GCU AUA AAG UAA CUG AGA CGG A-3′), and miR-340 mimics (5′-UCC GUC UCA GUU ACU UUA UAG C-3′) using Lipofectamine 3000 (Invitrogen, USA). The sequences of negative control (NC) of miR-340 mimics and miR-340 inhibitors were 5′-UUC UCC GAA CGU GUC ACG UTT-3′ and 5′-TTA AAC GTG TGT CGT ACT GAA-3′, respectively. The negative control of siRNA had a random sequence. To perform the interference of lncRNA, the CASC19 smart silencer (siCASC19) (RiboBio, Guangzhou, China) containing three siRNA sequences (5′-TGC TGG TAC CAC CTG CTT AC-3′; 5′-CAA AGA ACA GGA GAA CAC TC-3′; and 5′-TGC ATG CTT CTG ATG TGA GT-3′) and three antisense oligonucleotides (ASO) sequences (5′-CAC TAA CAA AGT TGA CCT T-3′; 5′-GAA TTG GAG TGC CTG GGT-3′; and 5′-CAT TCA AGG TGC TGT CCA A-3′) were transferred into cells.

### 4.10. Autophagic Flux Assay

Briefly, 3  × 10^5^ cells per well were incubated in a 24-well plate overnight. Then, cells were transfected with mRFP-GFP-LC3 double-labeled adenovirus (Hanbio, Shanghai, China) to label autophagosomes. After 2 h of transfection, the cells were washed twice with pre-cooled PBS and stained with DAPI. Intracellular autophagy was observed using a high-content imaging system (ImageXpress Micro 4, Molecular Devices, San Francisco, CA, USA). Double labeling of LC3 (green) and mRFP (red) immunofluorescence corresponds to the changes in the autophagic flux. After merging, the yellow puncta in the cell image symbolize the autophagosomes.

### 4.11. Dual-Luciferase Reporter Assay

The CASC19 luciferase reporter vector harboring miR-340-3p binding sequence (CASC19-WT) and site-directed mutation of targeted sequence (CASC19-MUT), and the FKBP5 3′-untranslated region (3′-UTR) luciferase reporter vector containing miR-340-3p targeted sites (FKBP5-WT) and site-directed mutation of targeted sequence (FKBP5-MUT) were constructed according to manufacturer’s instructions (Genomeditech, Shanghai, China). To confirm the targeting relationship between CASC19 and miR-340-3p, the cells under 50% confluence were transfected with the CASC19-WT or CASC19-MUT vector together with miR-340-3p mimics or miR-NC mimics. To detect whether FKBP5 is a direct target of miR-340-3p, the FKBP5-WT or FKBP5-MUT vector was transfected into cells together with miR-340-3p mimics or miR-NC mimics. After 24 h transfection, cell lysates were prepared, and the luciferase activity was measured using a dual-luciferase reporter kit (Promega, Madison, WI, USA) following the manufacturer’s instructions.

### 4.12. Statistical Analysis

Data are presented as means ± SD of at least three independent experiments. Data were analyzed with Student’s *t*-test or one-way analysis of variance (ANOVA) using SPSS 19.0 software (SPSS, Chicago, IL, USA). *p* < 0.05 indicated a significant difference between the indicated samples.

## Data Availability

The data supporting the present study are available from the corresponding author upon reasonable request.

## Supplementary Materials

The following supporting information can be downloaded at: https://www.mdpi.com/article/10.3390/ijms24033047/s1.

## Abbreviations

NPC: nasopharyngeal carcinoma; ceRNA: competing endogenous RNA; RNA-seq: RNA sequencing; TMT: tandem mass tag; ncRNAs: non-coding RNAs; DElncRNAs: differentially expressed lncRNAs; DEmRNAs: differentially expressed mRNAs; DEmiRNAs: differentially expressed miRNAs; WT: wild type; MUT: mutant type; HNSC: head and neck squamous cell carcinoma; DEGs: differentially expressed genes.
